# Square Root Statistics of Density Matrices and Their Applications

**DOI:** 10.3390/e26010068

**Published:** 2024-01-12

**Authors:** Lyuzhou Ye, Youyi Huang, James C. Osborn, Lu Wei

**Affiliations:** 1 Department of Computer Science, Texas Tech University, Lubbock, TX 79409, USA; lye@ttu.edu (L.Y.); youhuang@ttu.edu (Y.H.); 2 Computational Science Division, Argonne National Laboratory, Argonne, IL 60439, USA; osborn@anl.gov

**Keywords:** quantum entanglement, negativity, fidelity, Bures–Hall ensemble, random matrix theory

## Abstract

To estimate the degree of quantum entanglement of random pure states, it is crucial to understand the statistical behavior of entanglement indicators such as the von Neumann entropy, quantum purity, and entanglement capacity. These entanglement metrics are functions of the spectrum of density matrices, and their statistical behavior over different generic state ensembles have been intensively studied in the literature. As an alternative metric, in this work, we study the sum of the square root spectrum of density matrices, which is relevant to negativity and fidelity in quantum information processing. In particular, we derive the finite-size mean and variance formulas of the sum of the square root spectrum over the Bures–Hall ensemble, extending known results obtained recently over the Hilbert–Schmidt ensemble.

## 1. Introduction and Main Results

The understanding of entanglement is crucial to any successful quantum information processing task. In studying the degree of entanglement, researchers commonly employ entropy-based measures, for example, the von Neumann entropy [1] and quantum purity [2]. Additionally, various other entanglement metrics have been investigated, such as the entanglement capacity proposed in [3] as a quantum analogy to the heat capacity of classical systems. In the past decades, there has been considerable efforts in discovering the statistical behavior of the degree entanglement of quantum bipartite systems. These studies focus on computing the moments of the entanglement measures over different generic (pure) state models: the Hilbert–Schmidt ensemble, the Bures–Hall ensemble, and the emerging fermionic Gaussian ensemble. In the present work, we study the statistical behavior of the metric—the sum of the square root of the spectrum of density matrices over the Bures–Hall ensemble. The proposed metric is what we refer to as a square root statistic and is relevant to the negativity introduced in [4], a computable measure of entanglement between the subsystems of quantum bipartite models. Our primary findings are the exact formulas of the first two moments of the square root statistic. Moreover, the obtained formulas extend the recent the results of negativity [5] and fidelity [6] over the Hilbert–Schmidt ensemble to the Bures–Hall ensemble.

### 1.1. Square Root Spectrum and Applications

The sum of the square root of the spectrum of density matrices is defined as

(1) $$\Lambda =\sum_{i=1}^{m}\lambda_{i}^{\frac{1}{2}},$$

where *m* is the dimension of the density matrix and the set ${\left\{\lambda_{i}\right\}}_{i=1}^{m}$ is its spectrum. The random variable (1) is closely related to the negativity (2) and fidelity (3) as discussed below.

For a pure bipartite state $\rho =|\psi \rangle \langle \psi |$ with $|\psi \rangle =\sum_{j=1}^{m}\sqrt{\lambda_{j}}|jj\rangle$ and $\sum_{j=1}^{m}\lambda_{j}=1$ , where $|jj\rangle$ are the eigenvectors corresponding to the Schmidt coefficients, the negativity is defined as

(2) $$N\left(\rho \right)=\frac{\left|\right|\rho^{T_{A}}{\left|\right|}_{1}-1}{2}=\frac{\sum_{j\neq k}\sqrt{\lambda_{j}\lambda_{k}}}{2}=\frac{{\left(\sum_{j=1}^{m}\sqrt{\lambda_{j}}\right)}^{2}-1}{2}=\frac{\Lambda^{2}-1}{2},$$

where $\left|\right|\cdot {\left|\right|}_{1}$ is the trace norm (also known as the Schatten 1-norm) and $\rho^{T_{A}}$ refers to the partial transpose of ρ . Among different entanglement measures, the negativity possesses a unique property [7]. Assuming E(ρ) to be a weak entanglement monotone, characterized as a symmetric function of negative eigenvalues of $\rho^{T_{A}}$ , then E(ρ) is a non-decreasing function of N(ρ) . In the case that it is additive, it follows that E(ρ)=clog(1+2N(ρ)) for some constant c≥0 .

Fidelity [8] is a measure of the similarity between two quantum states. It quantifies how closely one quantum state resembles another. Given two quantum states characterized by the respective density matrices, σ and ρ , the fidelity is

(3) $$F\left(\sigma ,\rho \right)={\left(\mathrm{tr}\sqrt{\sqrt{\sigma }\rho \sqrt{\sigma }}\right)}^{2}.$$

In this work, we study the case

(4) $$\sigma =\frac{1}{m}I_{m},$$

which represents the maximum mixed state, and ρ is the random density matrix that corresponds to the Bures–Hall ensemble. In this case, we have

(5) $$F\left(\sigma ,\rho \right)=\frac{1}{m}\Lambda^{2}.$$

### 1.2. Description of Bures–Hall Ensemble

The Bures–Hall ensemble is introduced in the following (see also [9,10] for detailed formulations). Consider a bipartite system composed of two subsystems *A* and *B* of Hilbert space (complex vector space) with dimensions *m* and *n*, respectively. The Hilbert space $H_{A+B}=H_{A}⊗H_{B}$ . Let $|i^{A}\rangle$ and $|j^{B}\rangle$ be the complete basis of $H_{A}$ and $H_{B}$ . A random pure state of the composite system $H_{A+B}$ is defined as a linear combination of the basis $|i^{A}\rangle$ and $|j^{B}\rangle$ [9] as

(6) $$|\psi \rangle =\sum_{i=1}^{m}\sum_{j=1}^{n}z_{i,j}|i^{A}\rangle ⊗|j^{B}\rangle ,$$

where the coefficients $z_{i,j}$ are uniformly distributed over all possible values satisfying the constraint $\sum_{i=1}^{m}\sum_{j=1}^{n}{\left|z_{i,j}\right|}^{2}=1$ . We now consider a superposition of the state (6),

(7) $$|\varphi \rangle ∼|\psi \rangle +\left(U⊗I_{n}\right)|\psi \rangle ,$$

where U is an m×m unitary random matrix with the measure proportional to $\det{\left(I_{m}+U\right)}^{2\alpha +1}$ [11] with the parameter α taking half-integer values

(8) $$\alpha =n-m-\frac{1}{2}.$$

The corresponding density matrix of the pure state (7) is

(9) $$\rho =|\varphi \rangle \langle \varphi |,$$

with the probability constraint

(10) tr(ρ)=1,

which has been discussed in detail in [9]. We assume that m≤n without loss of generality. By partial tracing (purification) of the full density matrix (9) over the other subsystem *B* (environment), the reduced density matrix $\rho_{A}$ of the smaller subsystem *A* is obtained as

(11) $$\rho_{A}={\mathrm{tr}}_{B}\rho .$$

The density of the eigenvalues of $\rho_{A}$ ( $\lambda_{i}\in \left[0,1\right],i=1,\cdots ,m$ ) is the (generalized) complex Bures–Hall measure [7,12,13,14],

(12) $$f\left(\lambda \right)=\frac{1}{C}\delta \left(1-\sum_{i=1}^{m}\lambda_{i}\right)\prod_{1\leq i<j\leq m}\frac{{\left(\lambda_{i}-\lambda_{j}\right)}^{2}}{\lambda_{i}+\lambda_{j}}\prod_{i=1}^{m}\lambda_{i}^{\alpha },$$

where the constant *C* is

(13) $$C=\frac{2^{-m(m+2\alpha )}\pi^{m/2}}{\Gamma (m(m+2\alpha +1)/2)}\prod_{i=1}^{m}\frac{\Gamma (i+1)\Gamma (i+2\alpha +1)}{\Gamma \left(i+\alpha +\frac{1}{2}\right)}.$$

### 1.3. Main Results

We now introduce our main results of the first two moments of the statistic Λ , which are presented in Propositions 1 and 2 below.

**Proposition** **1.** *The exact mean of the square root statistic *Λ* in (1), valid for any subsystem dimensions m≤n under the Bures–Hall ensemble (12), is given by*

(14) $$\begin{array}{cc} E_{f}\left[\Lambda \right] & =\frac{\Gamma (d)}{\Gamma (d+\frac{1}{2})\pi }\sum_{k=0}^{m-1}\frac{\Gamma \left(k+2\alpha +m+2\right)\Gamma \left(m-k-\frac{1}{2}\right)\Gamma \left(k+\frac{3}{2}\right)}{\Gamma \left(k+2\alpha +m+\frac{5}{2}\right)\Gamma \left(m-k\right)\Gamma \left(k+1\right)}\\ \; & \times \frac{\Gamma \left(k+2\alpha +\frac{5}{2}\right)\Gamma \left(k+\alpha +\frac{5}{2}\right)}{\Gamma (k+2\alpha +2)\Gamma (k+\alpha +2)}\left(1+\frac{k+\alpha +1}{k+\alpha +\frac{3}{2}}\right), \end{array}$$

*where d is*

(15) $$d=\frac{1}{2}m\left(m+2\alpha +1\right).$$

**Proposition** **2.** *The exact second moment of the square root statistic *Λ* in (1), valid for any subsystem dimensions m≤n under the Bures–Hall ensemble (12), is given by*

(16) $$\begin{array}{cc} E_{f}\left[\Lambda^{2}\right] & =\frac{1}{\pi^{2}d}\sum_{k=0}^{m-1}\sum_{j=0}^{m-1}\frac{l_{k,0}l_{j,0}}{l_{k,\frac{1}{2}}l_{j,\frac{1}{2}}}(\left(2+\frac{1}{2(j+\alpha +1)}\right)\left(2+\frac{1}{2(k+\alpha +1)}\right)\\ \; & \;\;\;\;-\frac{1}{2\left(k-j-\frac{1}{2}\right)\left(j-k-\frac{1}{2}\right)}\left(1+\frac{j+\alpha +\frac{3}{2}}{j+\alpha +1}\frac{k+\alpha +\frac{3}{2}}{k+\alpha +1}\right)\\ \; & \;\;\;\;+\frac{\frac{3}{2}+j+\alpha }{\left(2+j+k+2\alpha )(3+j+k+2\alpha )(1+\alpha +j\right)})+1, \end{array}$$

*where d is denoted in (15), and*

(17) $$l_{k,\beta }=\frac{\Gamma (m+2\alpha +k+2+\beta )}{\Gamma (k+1+\beta )\Gamma (k+\alpha +1+\beta )\Gamma (k+2\alpha +2+\beta )\Gamma (m-k-\beta )}.$$

The proof of Proposition 1 and Proposition 2 are given, respectively, in Section 2.2 and Section 2.3. Moreover, the mean value of negativity (2) and fidelity (5), valid for any subsystem dimensions m≤n , are obtained as

(18) $$\begin{array}{c} E_{f}\left[N\right]=\frac{1}{2}\left(E_{f}\left[\Lambda^{2}\right]-1\right),\\ E_{f}\left[F\right]=\frac{1}{m}E_{f}\left[\Lambda^{2}\right], \end{array}$$

where the expectation $E_{f}\left[.\right]$ is taken over the Bures–Hall ensemble (12). By definition, the exact variance of Λ under the Bures–Hall ensemble is given by

(19) $$V_{f}\left[\Lambda \right]=E_{f}\left[\Lambda^{2}\right]-E_{f}^{2}\left[\Lambda \right].$$

**Figure 1:**
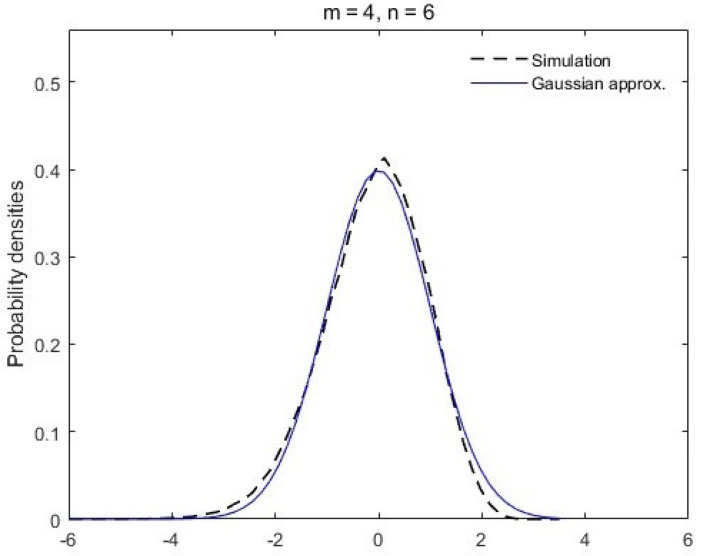
Probability density of *Y* in (20) in comparison with the Gaussian density. The dashed black line is plotted by the simulation results of *Y* with subsystem dimensions m=4 , n=6 and the solid blue line is the standard Gaussian density.

**Figure 2:**
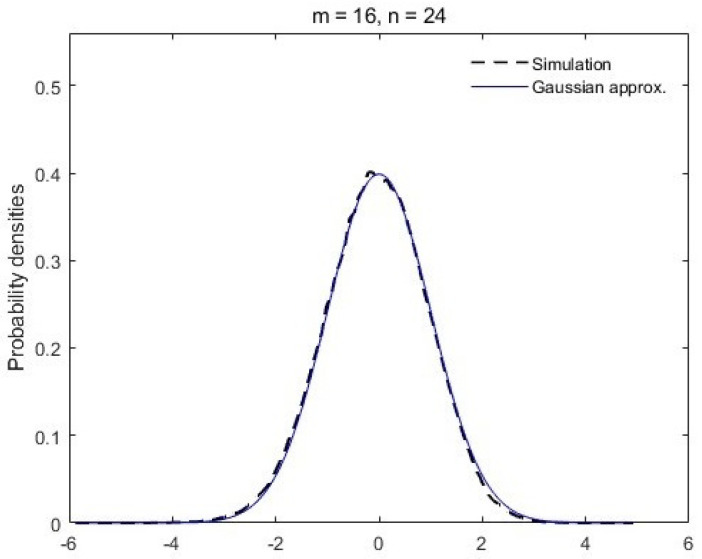
Probability density of *Y* in (20) versus the Gaussian density. The dashed black line is plotted by the simulation results of *Y* with subsystem dimensions m=16 , n=24 and the solid blue line is the standard Gaussian density.

With the obtained expressions of the mean (14) and variance (19), we can now study the distribution of Λ by standardizing it as

(20) $$Y=\frac{\Lambda -E_{f}\left[\Lambda \right]}{\sqrt{V_{f}\left[\Lambda \right]}},$$

where the standardized variable *Y* is supported in Y∈(−∞,∞) with zero mean and unit variance. As inspired by the Gaussian limit conjecture of von Neumann entropy [15,16], we plot, in Figure 1 and Figure 2, the simulation results of the standardized random valuable *Y* in comparison with the Gaussian density. It turns out that the distribution of Λ , similar to the von Neumann entropy, also approaches a Gaussian distribution when the subsystem dimensions increase with a fixed ratio m/n .

## 2. Moments Computation

In this section, we discuss the moment computation that gives rise to the results in Propositions 1 and 2. Specifically, in Section 2.1, we relate the computation of the moments to that over a more convenient ensemble with no fixed trace constraint. The detailed derivation of the first and second moments of the square root statistic Λ are presented in Section 2.2 and Section 2.3, respectively.

### 2.1. Ensemble Conversion

We calculate the random variable under the original ensemble (12) by converting it to an unconstrained ensemble of the Bures–Hall measure,

(21) $$h\left(x\right)=\frac{1}{C^{\prime }}\prod_{1\leq i<j\leq m}\frac{{\left(x_{i}-x_{j}\right)}^{2}}{x_{i}+x_{j}}\prod_{i=1}^{m}x_{i}^{\alpha }e^{-x_{i}},\;x_{i}\in \left[0,\infty \right)$$

where the constant $C^{\prime }$ depends on the constant *C* in (13) as

(22) $$C^{\prime }=C\Gamma \left(d\right)$$

with *d* denoting

(23) $$d=\frac{1}{2}m\left(m+2\alpha +1\right).$$

The density of the trace

(24) $$\theta =\sum_{i=1}^{m}x_{i},\;\theta \in \left[0,\infty \right)$$

is

(25) $$g\left(\theta \right)=\int_{x}h\left(x\right)\delta \left(\theta -\sum_{i=1}^{m}x_{i}\right)\prod_{i=1}^{m}\;dx_{i},$$

where, by the change of variables,

(26) $$x_{i}=\theta \lambda_{i},$$

we have

(27) $$\begin{array}{cc} g(\theta )\; & =\frac{C}{C^{\prime }}e^{\theta }\theta^{d-1}\int_{\lambda }f\left(\lambda \right)\;d\lambda_{i}\\ \; & =\frac{1}{\Gamma (d)}e^{-\theta }\theta^{d-1}. \end{array}$$

Keeping in mind the above result (27), the change of variables (26) in (12) now leads to the relation

(28) $$h\left(x\right)\prod_{i=1}^{m}dx_{i}=g\left(\theta \right)f\left(\lambda \right)d\theta \prod_{i=1}^{m}\;d\lambda_{i},$$

which implies that θ is independent of each $\lambda_{i}$ , i=1,⋯,m since the densities factorize. This fact allows us to relate the moments of

(29) $$\Lambda =\sum_{i=1}^{m}\lambda_{i}^{\frac{1}{2}}$$

over the Bures–Hall ensemble (12) to that of a random variable

(30) $$X=\sum_{i=1}^{m}x_{i}^{\frac{1}{2}}$$

over the unconstrained ensemble (21).

We now derive the relations between the first two moments of the random variables. For the first moment, by definition, we have

(31) $$\begin{array}{cc} E_{f}\left[\Lambda \right] & =\int_{0}^{\infty }\frac{e^{-\theta }\theta^{d-\frac{1}{2}}}{\Gamma (d+\frac{1}{2})}\;d\theta \int_{0}^{\infty }\Lambda f\left(\lambda \right)\prod_{i=1}^{m}\;d\lambda_{i}, \end{array}$$

where we have multiplied a constant

(32) $$1=\int_{0}^{\infty }\frac{1}{\Gamma (d+\frac{1}{2})}e^{-\theta }\theta^{d-\frac{1}{2}}\;d\theta$$

by using the result (27). In (31), substituting Λ with *X* gives

(33) $$\begin{array}{cc} E_{f}\left[\Lambda \right] & =\int_{0}^{\infty }\int_{0}^{\infty }\frac{X}{\theta^{\frac{1}{2}}}\frac{e^{-\theta }\theta^{d-\frac{1}{2}}}{\Gamma (d+\frac{1}{2})}f\left(\lambda \right)\;d\theta \prod_{i=1}^{m}\;d\lambda_{i}\\ \; & =\frac{\Gamma (d)}{\Gamma (d+\frac{1}{2})}\int_{0}^{\infty }g\left(\theta \right)\;d\theta \int_{0}^{\infty }Xf\left(\lambda \right)\prod_{i=1}^{m}\;d\lambda_{i}\\ \; & =\frac{\Gamma (d)}{\Gamma (d+\frac{1}{2})}\int_{0}^{\infty }Xh\left(x\right)\prod_{i=1}^{m}\;dx_{i}\\ \; & =\frac{\Gamma (d)}{\Gamma (d+\frac{1}{2})}E_{h}\left[X\right]. \end{array}$$

Similarly, we obtain the relation between the second moments as

(34) $$\begin{array}{c} E_{f}\left[\Lambda^{2}\right]=\frac{1}{d}E_{h}\left[X^{2}\right]. \end{array}$$

Using the result (33) and (34), we have

(35) $$\begin{array}{cc} V_{f}\left[\Lambda \right] & =E_{f}\left[\Lambda^{2}\right]-E_{f}^{2}\left[\Lambda \right]\\ \; & =\frac{1}{d}E_{h}\left[X^{2}\right]-{\left(\frac{\Gamma (d)}{\Gamma (d+\frac{1}{2})}\right)}^{2}E_{h}^{2}\left[X\right]. \end{array}$$

Therefore, the remaining task in obtaining the main results (14) and (16) is to calculate the first two moments of the statistic *X* in (30).

### 2.2. Calculation of the First Moment

Computing the average value of *X* requires the one-point correlation function of the unconstrained ensemble (21), which is [17]

(36) $$h_{1}\left(x\right)=\frac{1}{2m}\left(K_{01}\left(x,x\right)+K_{10}\left(x,x\right)\right),$$

where the correlation kernels $K_{01}\left(x,x\right)$ and $K_{10}\left(x,x\right)$ admit the following integral representations

(37) $$\begin{array}{cc} \; & K_{01}\left(x,y\right)=x^{2\alpha +1}\int_{0}^{1}t^{2\alpha +1}H_{\alpha }\left(ty\right)G_{\alpha +1}\left(tx\right)dt,\\ \; & K_{10}\left(x,y\right)=y^{2\alpha +1}\int_{0}^{1}t^{2\alpha +1}H_{\alpha +1}\left(tx\right)G_{\alpha }\left(ty\right)dt \end{array}$$

with

(38) $$\begin{array}{cc} \; & H_{q}\left(x\right)=G_{2,3}^{\;1,1}(\begin{array}{c} -m-2\alpha -1;m\\ 0;-q,-2\alpha -1 \end{array}|x)\equiv G_{2,3}^{1,1}\left(q|x\right)\\ \; & G_{q}\left(x\right)=G_{2,3}^{\;2,1}(\begin{array}{c} -m-2\alpha -1;m\\ 0,-q;-2\alpha -1 \end{array}|x)\equiv G_{2,3}^{2,1}\left(q|x\right) \end{array}$$

further denoting some Meijer G-functions [18]. The mean value of *X* is

(39) $$\begin{array}{cc} E_{h}\left[X\right] & =-m\int_{0}^{\infty }x^{\frac{1}{2}}h_{1}\left(x\right)\;dx\\ \; & =-\frac{1}{2}\int_{0}^{\infty }x^{\frac{1}{2}}\left(K_{01}\left(x,x\right)+K_{10}\left(x,x\right)\right)\;dx\\ \; & =-\frac{1}{2}\int_{1}^{\infty }\left(I_{\alpha }^{\frac{1}{2}}\left(t\right)+I_{\alpha +1}^{\frac{1}{2}}\left(t\right)\right)\;dt, \end{array}$$

where we have used the notation [19], Equation (31)

(40) $$I_{q}^{\left(\beta \right)}\left(t\right)=\int_{0}^{\infty }x^{\beta }G_{2,3}^{1,1}\left(q|tx\right)G_{2,3}^{2,1}\left(q|tx\right)dx,\;t>0.$$

The above integration has been evaluated in [19] as

(41) $$I_{q}^{\left(\beta \right)}\left(t\right)=t^{-\beta -1}I_{q}^{\left(\beta \right)},$$

with $I_{q}^{\left(\beta \right)}$ denoting the *t* independent part

(42) $$\begin{array}{cc} I_{q}^{\left(\beta \right)} & =\sum_{k=0}^{m-1}\frac{{\left(-1\right)}^{k}\Gamma \left(k+2\alpha +m+2\right)\Gamma \left(m-k-\beta \right)}{\Gamma (k+2\alpha +2)\Gamma (k+2\alpha +2-q)\Gamma (m-k)k!}\\ \; & \;\;\;\;\times \frac{\Gamma (k+\beta +2\alpha +2)\Gamma (k+\beta +2\alpha +2-q)}{\Gamma (k+\beta +2\alpha +m+2)\Gamma (-k-\beta )}. \end{array}$$

Inserting the above result (42) into (39) and evaluating the integration over *t*, one obtains

(43) $$\begin{array}{cc} E_{h}\left[X\right] & =-\sum_{k=0}^{m-1}\frac{{\left(-1\right)}^{k}\Gamma \left(k+2\alpha +m+2\right)\Gamma \left(m-k-\frac{1}{2}\right)}{\Gamma (k+2\alpha +2)\Gamma (k+\alpha +2)\Gamma (m-k)k!}\frac{\Gamma \left(k+\frac{1}{2}+2\alpha +2\right)\Gamma \left(k+\frac{1}{2}+\alpha +2\right)}{\Gamma \left(k+\frac{1}{2}+2\alpha +m+2\right)\Gamma \left(-k-\frac{1}{2}\right)}\\ & \;\;\;\;-\sum_{k=0}^{m-1}\frac{{\left(-1\right)}^{k}\Gamma \left(k+2\alpha +m+2\right)\Gamma \left(m-k-\frac{1}{2}\right)}{\Gamma (k+2\alpha +2)\Gamma (k+\alpha +1)\Gamma (m-k)k!}\frac{\Gamma \left(k+\frac{1}{2}+2\alpha +2\right)\Gamma \left(k+\frac{1}{2}+\alpha +1\right)}{\Gamma \left(k+\frac{1}{2}+2\alpha +m+2\right)\Gamma \left(-k-\frac{1}{2}\right)}. \end{array}$$

By using the following identity of gamma function

(44) $$\Gamma \left(-\frac{1}{2}-k\right)={\left(-1\right)}^{k-1}\frac{\Gamma \left(\frac{1}{2}\right)\Gamma \left(\frac{1}{2}\right)}{\Gamma (k+1+\frac{1}{2})},$$

the mean value is further simplified to

(45) $$\begin{array}{cc} E_{h}\left[X\right]= & \;\;\frac{1}{\pi }\sum_{k=0}^{m-1}\frac{\Gamma \left(k+2\alpha +m+2\right)\Gamma \left(m-k-\frac{1}{2}\right)\Gamma \left(k+\frac{3}{2}\right)}{\Gamma \left(k+2\alpha +m+\frac{5}{2}\right)\Gamma \left(m-k\right)\Gamma \left(k+1\right)}\frac{\Gamma \left(k+2\alpha +\frac{5}{2}\right)\Gamma \left(k+\alpha +\frac{5}{2}\right)}{\Gamma (k+2\alpha +2)\Gamma (k+\alpha +2)}\\ & \times \left(1+\frac{k+\alpha +1}{k+\alpha +\frac{3}{2}}\right). \end{array}$$

Inserting the result (45) into the relation (33), the first moment of Λ is obtained as

(46) $$\begin{array}{cc} E_{f}\left[\Lambda \right] & =\frac{\Gamma (d)}{\Gamma (d+\frac{1}{2})\pi }\sum_{k=0}^{m-1}\frac{\Gamma \left(k+2\alpha +m+2\right)\Gamma \left(m-k-\frac{1}{2}\right)\Gamma \left(k+\frac{3}{2}\right)}{\Gamma \left(k+2\alpha +m+\frac{5}{2}\right)\Gamma \left(m-k\right)\Gamma \left(k+1\right)}\frac{\Gamma \left(k+2\alpha +\frac{5}{2}\right)\Gamma \left(k+\alpha +\frac{5}{2}\right)}{\Gamma (k+2\alpha +2)\Gamma (k+\alpha +2)}\\ & \;\;\;\;\times \left(1+\frac{k+\alpha +1}{k+\alpha +\frac{3}{2}}\right). \end{array}$$

This completes the proof of Proposition 1.

### 2.3. Calculation of the Second Moment

According to the relation of second moments (35), it now suffices to calculate $E_{h}\left[X^{2}\right]$ in obtaining $E_{h}\left[\Lambda^{2}\right]$ . By definition, we have

(47) $$\begin{array}{cc} E_{h}\left[X^{2}\right] & =\int_{x}{\left(\sum_{i=1}^{m}x_{i}^{\frac{1}{2}}\right)}^{2}h\left(x\right)\prod_{i=1}^{m}\;dx_{i}\\ & =\int_{x}\left(\sum_{i=1}^{m}x_{i}\right)h\left(x\right)\prod_{i=1}^{m}\;dx_{i}+2\int_{x}\left(\sum_{1\leq i<j\leq m}x_{i}^{\frac{1}{2}}x_{j}^{\frac{1}{2}}\right)h\left(x\right)\prod_{i=1}^{m}\;dx_{i}\\ \; & =m\int_{0}^{\infty }xh_{1}\left(x\right)\;dx+m\left(m-1\right)\int_{0}^{\infty }\int_{0}^{\infty }x^{\frac{1}{2}}y^{\frac{1}{2}}h_{2}\left(x,y\right)\;dx\;dy. \end{array}$$

To proceed the above integrals, one will need the joint density of one and two arbitrary eigenvalues, respectively, denoted by $h_{1}\left(x\right)$ and $h_{2}\left(x,y\right)$ . The former one is given in (36) and the latter one in [17,20]

(48) $$\begin{array}{cc} h_{2}\left(x,y\right)=\;\; & \frac{1}{4m(m-1)}(\left(K_{01}\left(x,x\right)+K_{10}\left(x,x\right)\right)\left(K_{01}\left(y,y\right)+K_{10}\left(y,y\right)\right)-2K_{01}\left(x,y\right)K_{01}\left(y,x\right)\\ & -2K_{10}\left(x,y\right)K_{10}\left(y,x\right)-2K_{00}\left(x,y\right)K_{11}\left(x,y\right)-2K_{00}\left(y,x\right)K_{11}\left(y,x\right)), \end{array}$$

where

(49) $$\begin{array}{cc} \; & K_{00}\left(x,y\right)=\int_{0}^{1}t^{2\alpha +1}H_{\alpha }\left(tx\right)H_{\alpha +1}\left(ty\right)\;dt\\ \; & K_{01}\left(x,y\right)=x^{2\alpha +1}\int_{0}^{1}t^{2\alpha +1}H_{\alpha }\left(ty\right)G_{\alpha +1}\left(tx\right)\;dt\\ \; & K_{10}\left(x,y\right)=y^{2\alpha +1}\int_{0}^{1}t^{2\alpha +1}H_{\alpha +1}\left(tx\right)G_{\alpha }\left(ty\right)\;dt\\ \; & K_{11}\left(x,y\right)={\left(xy\right)}^{2\alpha +1}\int_{0}^{1}t^{2\alpha +1}G_{\alpha +1}\left(tx\right)G_{\alpha }\left(ty\right)\;dt-\frac{x^{\alpha }y^{\alpha +1}}{x+y}. \end{array}$$

By using the densities (36) and (48), computing the integrals in (47) now boils down to computing

(50) $$E_{h}\left[X^{2}\right]=\frac{1}{2}I_{A}-\frac{1}{2}\left(I_{B}+I_{C}\right)-I_{D}+E_{h}^{2}\left[X\right],$$

where

(51) $$\begin{array}{cc} I_{A} & =\int_{0}^{\infty }x\left(K_{01}\left(x,x\right)+K_{10}\left(x,x\right)\right)\;dx\\ I_{B} & =\int_{0}^{\infty }\int_{0}^{\infty }x^{\frac{1}{2}}y^{\frac{1}{2}}K_{01}\left(x,y\right)K_{01}\left(y,x\right)\;dx\;dy\\ I_{C} & =\int_{0}^{\infty }\int_{0}^{\infty }x^{\frac{1}{2}}y^{\frac{1}{2}}K_{10}\left(x,y\right)K_{10}\left(y,x\right)\;dx\;dy\\ I_{D} & =\int_{0}^{\infty }\int_{0}^{\infty }x^{\frac{1}{2}}y^{\frac{1}{2}}K_{00}\left(x,y\right)K_{11}\left(x,y\right)\;dx\;dy. \end{array}$$

#### 2.3.1. Calculation of $I_{A}$

Using the same strategy in calculating $E_{h}\left[X\right]$ in Section 2.2 (see also [15], Equations (52)–(55)), for example, the integral $I_{A}$ in (51) is computed as

(52) $$\begin{array}{cc} I_{A}\; & =-\sum_{k=0}^{m-1}(\frac{{\left(-1\right)}^{k+m}\Gamma \left(k+2\alpha +m+2\right)\Gamma \left(k+2\right)}{\Gamma (k+2\alpha +2)\Gamma (k+\alpha +2)\Gamma (m-k)k!}\frac{\Gamma (k+2\alpha +3)\Gamma (k+\alpha +3)}{\Gamma (k+2\alpha +m+3)\Gamma (k-m+2)}\\ & \;\;\;\;+\frac{{\left(-1\right)}^{k+m}\Gamma \left(k+2\alpha +m+2\right)\Gamma \left(k+2\right)}{\Gamma (k+2\alpha +2)\Gamma (k+\alpha +1)\Gamma (m-k)k!}\frac{\Gamma (k+2\alpha +3)\Gamma (k+\alpha +2)}{\Gamma (k+2\alpha +m+3)\Gamma (k-m+2)})\\ \; & =m(2\alpha +m+1). \end{array}$$

#### 2.3.2. Calculation of $I_{B}$ and $I_{C}$

For the calculation of $I_{B}$ and $I_{C}$ , it is more convenient to use the finite sum representation [19,20] of the Meijer G-functions $G_{2,3}^{1,1}$ in the kernels (49) and evaluate the integrals over *t* by using the identity [18]

(53) $$\begin{array}{c} \int_{0}^{1}x^{a-1}G_{p,q}^{\;m,n}(\begin{array}{c} a_{1},\cdots ,a_{n};a_{n+1},\cdots ,a_{p}\\ b_{1},\cdots ,b_{m};b_{m+1},\cdots ,b_{q} \end{array}|\eta x)\;dx=G_{p+1,q+1}^{\;m,n+1}(\begin{array}{c} 1-a,a_{1},\cdots ,a_{n};a_{n+1},\cdots ,a_{p}\\ b_{1},\cdots ,b_{m};b_{m+1},\cdots ,b_{q},-a \end{array}|\eta ). \end{array}$$

Consequently, the integrals $I_{B}$ and $I_{C}$ are computed to

(54) $$\begin{array}{cc} \; & I_{B}=\sum_{j,k=0}^{m-1}f_{j,k}f_{k,j}\\ \; & I_{C}=\sum_{j,k=0}^{m-1}g_{j,k}g_{k,j}, \end{array}$$

where we denote

(55) $$f_{j,k}=\frac{{\left(-1\right)}^{j}\Gamma \left(m+2\alpha +j+2\right)}{\Gamma (j+1)\Gamma (\alpha +j+1)\Gamma (2\alpha +j+2)\Gamma (m-j)}\int_{0}^{\infty }x^{\frac{1}{2}}G_{3,4}^{\;2,2}(\begin{array}{c} j-k,j-m;m+2\alpha +j+1\\ 2\alpha +j+1,\alpha +j;j,j-k-1 \end{array}|x)\;dx$$

(56) $$g_{j,k}=\frac{{\left(-1\right)}^{j}\Gamma \left(m+2\alpha +j+2\right)}{\Gamma (j+1)\Gamma (\alpha +j+2)\Gamma (2\alpha +j+2)\Gamma (m-j)}\int_{0}^{\infty }x^{\frac{1}{2}}G_{3,4}^{\;2,2}(\begin{array}{c} j-k,j-m;m+2\alpha +j+1\\ 2\alpha +j+1,\alpha +j+1;j,j-k-1 \end{array}|x)\;dx.$$

Using the Mellin transform of the Meijer G-function [18]

(57) $$\int_{0}^{\infty }x^{s-1}G_{p,q}^{\;m,n}(\begin{array}{c} a_{1},\cdots ,a_{n};a_{n+1},\cdots ,a_{p}\\ b_{1},\cdots ,b_{m};b_{m+1},\cdots ,b_{q} \end{array}|\eta x)\;dx=\frac{\eta^{-s}\prod_{j=1}^{m}\Gamma \left(b_{j}+s\right)\prod_{j=1}^{n}\Gamma \left(1-a_{j}-s\right)}{\prod_{j=n+1}^{p}\Gamma \left(a_{j}+s\right)\prod_{j=m+1}^{q}\Gamma \left(1-b_{j}-s\right)},$$

the integrals in (55) and (56) are, respectively, calculated as

(58) $$\begin{array}{cc} f_{j,k} & =\frac{{\left(-1\right)}^{j}\Gamma \left(m+2\alpha +j+2\right)\Gamma \left(j+2\alpha +1+\frac{3}{2}\right)}{\Gamma (j+1)\Gamma (\alpha +j+1)\Gamma (2\alpha +j+2)\Gamma (m-j)}\\ & \;\;\;\;\times \frac{\Gamma \left(j+\alpha +\frac{3}{2}\right)\Gamma \left(1-j+k-\frac{3}{2}\right)\Gamma \left(1-j+m-\frac{3}{2}\right))}{\Gamma \left(m+2\alpha +j+\frac{5}{2}\right)\Gamma \left(-\frac{1}{2}-j\right)\Gamma \left(\frac{1}{2}-j+k\right)} \end{array}$$

and

(59) $$g_{j,k}=f_{j,k}\frac{j+\alpha +\frac{3}{2}}{j+\alpha +1}.$$

Applying the identity of Gamma function (44), $f_{j,k}$ is now written as

(60) $$\begin{array}{cc} f_{j,k} & =-\frac{\Gamma \left(m+2\alpha +j+2\right)\Gamma \left(j+\frac{3}{2}\right)\Gamma \left(m-j-\frac{1}{2}\right)}{\Gamma (j+1)\Gamma (j+\alpha +1)\Gamma (j+2\alpha +2)\Gamma (m-j)}\frac{\Gamma \left(j+2\alpha +\frac{5}{2}\right)\Gamma \left(j+\alpha +\frac{3}{2}\right)}{\Gamma \left(m+2\alpha +j+\frac{5}{2}\right)\left(k-j-\frac{1}{2}\right)\pi }\\ \; & =-\frac{1}{\pi }\frac{l_{j,0}}{l_{j,\frac{1}{2}}}\frac{1}{k-j-\frac{1}{2}}, \end{array}$$

where we have utilized the shorthand notation

(61) $$l_{j,x}=\frac{\Gamma (m+2\alpha +j+2+x)}{\Gamma (j+1+x)\Gamma (j+\alpha +1+x)\Gamma (j+2\alpha +2+x)\Gamma (m-j-x)}.$$

#### 2.3.3. Calculation of $I_{D}$

To calculate $I_{D}$ , we use another form of the correlation kernels [18]

(62) $$\begin{array}{cc} \; & K_{00}\left(x,y\right)=\sum_{k=0}^{m-1}p_{k}\left(x\right)q_{k}\left(y\right)\\ \; & K_{11}\left(x,y\right)=x^{\alpha }y^{\alpha +1}e^{-x-y}\sum_{k=0}^{m-1}P_{k}\left(-y\right)Q_{k}\left(-x\right)-w\left(x,y\right), \end{array}$$

with the weight function w(x,y) of the biorthogonal polynomials $p_{k}\left(x\right),q_{l}\left(y\right)$ ,

(63) $$\int_{0}^{\infty }\int_{0}^{\infty }p_{k}\left(x\right)q_{l}\left(y\right)w\left(x,y\right)dxdy=\delta_{kl},$$

given by

(64) $$w\left(x,y\right)=\frac{x^{\alpha }y^{\alpha +1}e^{-x-y}}{x+y}.$$

The functions in (62) can be expressed via Meijer G-functions [17,20] as

(65) $$\begin{array}{cc} p_{j}\left(x\right) & =\sqrt{2}{\left(-1\right)}^{j}G_{2,3}^{\;1,1}\left(\begin{array}{c} -2\alpha -1-j;j+1\\ 0;-\alpha ,-2\alpha -1 \end{array}|x\right)\\ q_{j}\left(x\right) & =\sqrt{2}{\left(-1\right)}^{j}\left(j+\alpha +1\right)G_{2,3}^{\;1,1}\left(\begin{array}{c} -2\alpha -1-j;j+1\\ 0;-\alpha -1,-2\alpha -1 \end{array}|x\right)\\ P_{j}\left(x\right) & =\sqrt{2}{\left(-1\right)}^{j+1}e^{-x}G_{2,3}^{\;2,1}\left(\begin{array}{c} -\alpha -j-1;\alpha +j+1\\ 0,\alpha ;-\alpha -1 \end{array}|-x\right)\\ Q_{j}\left(x\right) & =\sqrt{2}{\left(-1\right)}^{j+1}\left(j+\alpha +1\right)e^{-x}G_{2,3}^{\;2,1}(\begin{array}{c} -\alpha -j;\alpha +j+2\\ 0,\alpha +1;-\alpha \end{array}|-x). \end{array}$$

Using the representations (62), the corresponding integrals of $I_{D}$ in (51) are now written as

(66) $$\begin{array}{cc} I_{D} & =\sum_{j,k=0}^{m-1}\int_{0}^{\infty }\int_{0}^{\infty }x^{\frac{1}{2}}x^{\alpha }e^{-x}p_{j}\left(x\right)Q_{k}\left(-x\right)y^{\frac{1}{2}}y^{\alpha +1}e^{-y}q_{j}\left(y\right)P_{k}\left(-y\right)\;dx\;dy\\ \; & \;\;\;\;-\sum_{j=0}^{m-1}\int_{0}^{\infty }\int_{0}^{\infty }x^{\frac{1}{2}}y^{\frac{1}{2}}p_{j}\left(x\right)q_{j}\left(y\right)\frac{x^{\alpha }y^{\alpha +1}e^{-x-y}}{x+y}\;dx\;dy. \end{array}$$

In (66), the first double integral can be separately evaluated over *x* and *y* by the Formula (57). Explicitly, for the integration over *x*, we have

(67) $$\begin{array}{cc} \; & \int_{0}^{\infty }x^{\beta }x^{\alpha }e^{-x}p_{j}\left(x\right)Q_{k}\left(-x\right)dx\\ & ={\left(-1\right)}^{j+k+1}\int_{0}^{\infty }G_{2,3}^{\;1,1}(\begin{array}{c} -2\alpha -1-j;j+1\\ 0;-\alpha ,-2\alpha -1 \end{array}|x)G_{2,3}^{\;2,1}(\begin{array}{c} \beta -k;\beta +2\alpha +k+2\\ \beta +\alpha ,\beta +2\alpha +1;\beta \end{array}|x)\;dx\\ \; & ={\left(-1\right)}^{j+k+1}\int_{0}^{\infty }{\frac{\Gamma (2\alpha +2+j)}{\Gamma (1+\alpha )\Gamma (2\alpha +2)\Gamma (j+1)}}_{2}F_{2}\left(2\alpha +2+j,-j;1+\alpha ,2\alpha +2;x\right)\\ \; & \;\;\;\;\times G_{2,3}^{\;2,1}(\begin{array}{c} \beta -k;\beta +2\alpha +k+2\\ \beta +\alpha ,\beta +2\alpha +1;\beta \end{array}|x)\;dx\\ \; & =\sum_{i=0}^{j}\frac{{\left(-1\right)}^{i+j+k+1}\Gamma \left(2\alpha +2+j+i\right)}{\Gamma (1+\alpha +i)\Gamma (2\alpha +2+i)\Gamma (i+1)\Gamma (j-i+1)}\int_{0}^{\infty }x^{i}G_{2,3}^{\;2,1}(\begin{array}{c} \beta -k;\beta +2\alpha +k+2\\ \beta +\alpha ,\beta +2\alpha +1;\beta \end{array}|x)\;dx\\ \; & =\sum_{i=0}^{j}\frac{{\left(-1\right)}^{i+j+1}\Gamma \left(2\alpha +2+j+i\right)\Gamma \left(\beta +i+1\right)}{\Gamma (1+\alpha +i)\Gamma (2\alpha +2+i)\Gamma (i+1)\Gamma (j-i+1)}\frac{\Gamma (\beta +\alpha +i+1)\Gamma (\beta +2\alpha +i+2)}{\Gamma (\beta +2\alpha +k+i+3)\Gamma (\beta +i+1-k)}. \end{array}$$

Similarly, for the integration over *y*, we have

(68) $$\begin{array}{cc} \; & \int_{0}^{\infty }y^{\beta }y^{\alpha +1}e^{-y}q_{j}\left(y\right)P_{k}\left(-y\right)\;dy\\ & =\sum_{s=0}^{j}\frac{{\left(-1\right)}^{s+j+1}\Gamma \left(2\alpha +2+j+s\right)\Gamma \left(\beta +s+1\right)}{\Gamma (2+\alpha +s)\Gamma (2\alpha +2+s)\Gamma (s+1)\Gamma (j-s+1)}\frac{\Gamma (\beta +\alpha +s+2)\Gamma (\beta +2\alpha +s+2)}{\Gamma (\beta +2\alpha +k+s+3)\Gamma (\beta +s+1-k)}. \end{array}$$

For the integral that involves the weight function w(x,y) in (66), we have

(69) $$\begin{array}{cc} \; & \int_{0}^{\infty }\int_{0}^{\infty }x^{\beta }y^{\beta }p_{j}\left(x\right)q_{j}\left(y\right)\frac{x^{\alpha }y^{\alpha +1}e^{-x-y}}{x+y}\;dx\;dy\\ \; & =\sum_{i=0}^{j}\sum_{k=0}^{j}\frac{1}{\Gamma (i+1)\Gamma (j-i+1)}\frac{\Gamma (\beta +\alpha +i+1)\Gamma (\beta +\alpha +k+2)}{2\beta +i+k+2\alpha +2}\\ & \;\;\;\;\times \frac{{\left(-1\right)}^{k+i}\Gamma \left(2\alpha +2+j+i\right)}{\Gamma (1+\alpha +i)\Gamma (2\alpha +2+i)}\frac{\Gamma (2\alpha +2+j+k)}{\Gamma (2+\alpha +k)\Gamma (2\alpha +2+k)\Gamma (k+1)\Gamma (j-k+1)}. \end{array}$$

Applying the results (67)–(69) with $\beta =\frac{1}{2}$ in (66), we obtain

(70) $$\begin{array}{cc} \; & I_{D}=-\frac{1}{\pi^{2}}\sum_{i=0}^{m-1}\sum_{j=0}^{m-1}\frac{l_{i,0}l_{j,0}}{l_{i,\frac{1}{2}}l_{j,\frac{1}{2}}}\frac{\frac{1}{2}+\alpha +j+1}{\left(2+i+j+2\alpha )(2+i+j+2\alpha +1)(1+\alpha +j\right)}, \end{array}$$

where we recall the function $l_{j,x}$ is denoted in (61).

Inserting the summation forms (45), (52), (54), and (70), we obtain the second moment of *X*

(71) $$\begin{array}{cc} E_{h}\left[X^{2}\right] & =\frac{m(2\alpha +m+1)}{2}+\frac{1}{\pi^{2}}\sum_{k=0}^{m-1}\sum_{j=0}^{m-1}\frac{l_{k,0}l_{j,0}}{l_{k,\frac{1}{2}}l_{j,\frac{1}{2}}}(\left(2+\frac{1}{2(j+\alpha +1)}\right)\left(2+\frac{1}{2(k+\alpha +1)}\right)\\ \; & \;\;\;\;-\frac{1}{2\left(k-j-\frac{1}{2}\right)\left(j-k-\frac{1}{2}\right)}\left(1+\frac{j+\alpha +\frac{3}{2}}{j+\alpha +1}\frac{k+\alpha +\frac{3}{2}}{k+\alpha +1}\right)\\ & \;\;\;\;+\frac{\frac{3}{2}+j+\alpha }{\left(2+j+k+2\alpha )(3+j+k+2\alpha )(1+\alpha +j\right)}). \end{array}$$

Now, using the relation (34), we complete the proof of Proposition 2.

## 3. Conclusions

In this work, we compute the exact mean values of negativity and fidelity over the Bures–Hall ensemble via computing the first two moments of the sum of the square root spectrum of density matrices. We derived the results by utilizing established formulas of the correlation functions of the Bures–Hall ensemble, along with corresponding tools of special functions. Future work will involve computing higher-order moments of the sum of the square root spectrum and determining its asymptotic distributions.

## Data Availability

Data is contained within the article.
